# Immune reconstitution inflammatory syndrome associated with dermatophytoses in two HIV-1 positive patients in rural Tanzania: a case report

**DOI:** 10.1186/s12879-016-1824-4

**Published:** 2016-09-20

**Authors:** Herry Mapesi, Adrià Ramírez, Marcel Tanner, Christoph Hatz, Emilio Letang, the KIULARCO Study Group, Aschola Asantiel, Manuel Battegay, Adolphina Chale, Diana Faini, Ingrid Felger, Gideon Francis, Hansjakob Furrer, Anna Gamell, Tracy Glass, Christoph Hatz, Specioza Hwaya, Bryson Kasuga, Namvua Kimera, Yassin Kisunga, Thomas Klimkait, Emilio Letang, Antonia Luhombero, Lameck B. Luwanda, Herry Mapesi, Leticia Mbwile, Mengi Mkulila, Julius Mkumbo, Margareth Mkusa, Dorcus K. Mnzava, Germana Mossad, Dolores Mpundunga, Athumani Mtandanguo, Kim D. Mwamelo, Selerine Myeya, Sanula Nahota, Regina Ndaki, Agatha Ngulukila, Alex John Ntamatungiro, Leila Samson, George Sikalengo, Marcel Tanner, Fiona Vanobberghen, Aneth V. Kalinjuma, Maja Weisser

**Affiliations:** 1 Chronic Diseases Clinic of Ifakara, Ifakara Health Institute, P. O Box 53, Ifakara, Tanzania; 2 University of Basel, Basel, Switzerland; 3 Chronic Diseases Clinic of Ifakara, Swiss Tropical & Public Health Institute (Swiss TPH), Ifakara Health Institute (IHI), Ifakara branch, P.O. Box 53, Ifakara, Tanzania; 4 University Hospital Son Espases, Palma de Mallorca, Spain; 5 ISGLOBAL, Barcelona Ctr. Int. Health Res. (CRESIB), Hospital Clínic-Universitat de Barcelona, Barcelona, Spain

**Keywords:** HIV, Immune reconstitution inflammatory syndrome, Case report

## Abstract

**Background:**

Immune reconstitution inflammatory syndrome associated with dermatophytoses (tinea-IRIS) may cause considerable morbidity. Yet, it has been scarcely reported and is rarely considered in the differential diagnosis of HIV associated cutaneous lesions in Africa. If identified, it responds well to antifungals combined with steroids. We present two cases of suspected tinea-immune reconstitution inflammatory syndrome from a large HIV clinic in rural Tanzania.

**Cases presentation:**

A first case was a 33 years-old female newly diagnosed HIV patient with CD4 count of 4 cells/μL (0 %), normal complete blood count, liver and renal function tests was started on co-formulated tenofovir/emtricitabine/efavirenz and prophylactic cotrimoxazole. Two weeks later she presented with exaggerated inflammatory hyperpigmented skin plaques with central desquamation, active borders and scratch lesions on the face, trunk and lower limbs. Tinea-IRIS was suspected and fluconazole (150 mg daily) and prednisolone (1 mg/Kg/day tapered down after 1 week) were given. Her symptoms subsided completely after 8 weeks of treatment, and her next CD4 counts had increased to 134 cells/μL (11 %). The second case was a 35 years-old female newly diagnosed with HIV. She had 1 CD4 cell/μL (0 %), haemoglobin 9.8 g/dl, and normal renal and liver function tests. Esophageal candidiasis and normocytic-normochromic anaemia were diagnosed. She received fluconazole, prophylactic cotrimoxazole and tenofovir/emtricitabine/efavirenz. Seven weeks later she presented with inflammatory skin plaques with elevated margins and central hyperpigmentation on the trunk, face and limbs in the frame of a good general recovery and increased CD4 counts (188 cells/μL, 6 %). Tinea-IRIS was suspected and treated with griseofulvin 500 mg daily and prednisolone 1 mg/Kg tapered down after 1 week, with total resolution of symptoms in 2 weeks.

**Conclusion:**

The two cases had advanced immunosuppression and developed de-novo exaggerated manifestation of inflammatory lesions compatible with tinea corporis and tinea facies in temporal association with antiretroviral treatment initiation and good immunological response. This is compatible with unmasking tinea-IRIS, and reminds African clinicians about the importance of considering this entity in the differential diagnosis of patients with skin lesions developing after antiretroviral treatment initiation.

## Background

Immune reconstitution inflammatory syndrome asociated with dermatophytoses (tinea-IRIS) may cause considerable morbidity given the high prevalence of fungal skin infections, especially in HIV/AIDS patients with late presentation. Despite having been reported to be associated with 52–78 % of cutaneous associated IRIS [1], it has been scarcely reported and is rarely considered in the differential diagnosis of HIV-associated cutaneous lesions in Sub-Saharan Africa (SSA). If identified, it responds well to antifungals combined with steroids [2]. We present two photographically documented cases of suspected tinea-IRIS in HIV-individuals in rural Tanzania. The two patients developed the lesions two and 7 weeks respectively after starting antiretroviral treatment (ART). They were treated with fluconazole and griseofulvin respectively combined with oral prednisolone. The symptoms resolved after 8 and 2 weeks in both patients respectively.

### Case I presentation

A 33 years-old Tanzanian woman presented to the HIV clinic suffering from skin itching for 3 months prior to the visit. She tested positive for HIV and she was enrolled in care. At the baseline physical examination she had pruritic papular eruptions (PPE) involving both upper and lower limbs. Other systems and the vital signs were unremarkable.

### Investigations

Her baseline investigations showed CD4 count of 4 cells/μL (0 %), with normal complete blood count (CBC), liver function test (LFT) and estimated glomerular filtration rate (eGFR). She had negative Cryptococcal plasma antigen (CRAG) and Venereal Disease Research Laboratory (VDRL) test. She also had negative Hepatitis B surface antigen (HBsAg) and negative screening test for cervical cancer screening.

### Treatment

She was started ART on day four after enrollment into care in our HIV-Clinic on co-formulated tenofovir disoproxil fumarate 300 mg/emtricitabine 200 mg/efavirenz 600 mg (TDF/FTC/EFV) with prophylactic cotrimoxazole, 960 mg once daily. She was also prescribed symptomatic treatment for PPE, receiving cetirizine 5 mg once daily for 1 week.

### Outcome and follow-Up

**Fig. 1:**
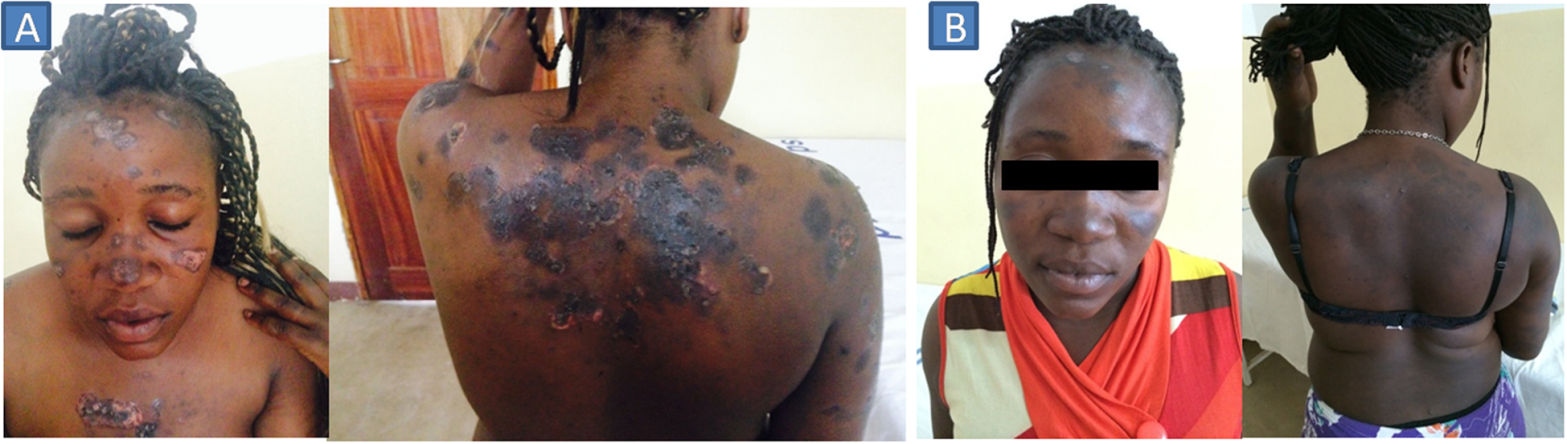
Pictures of the case I before and after treatment. Picture **a** shows exaggerated inflammatory manifestation, including hyperpigmented skin plaques with central desquamation and active borders. The lesions were itching in nature surrounded by scratchy lesions on the face, trunk and lower limbs. Picture **b** shows improvement after 8 weeks treatment with fluconazole, prednisolone and cetirizine

Two weeks after initiating ART she returned to the HIV-clinic with an exaggerated inflammatory manifestation, including hyperpigmented skin plaques with central desquamation and active borders on the face, trunk and lower limbs. The lesions were itching and surrounded by scratchy lesions (Fig. 1). Tinea-IRIS was suspected and fluconazole (150 mg daily), prednisolone (1 mg/kg for 1 weeks tapered down during the second week) and cetirizine 5 mg once daily for 1 week were given. Her symptoms subsided after 8 weeks of antifungal treatment (Fig. 1). Her CD4 count after 2 months on ART increased to 134 cells/μL (11 %) (Table 1).

**Table 1:** Summary of clinical presentation, treatment and outcome of both patients

Patient	Initial Diagnosis	Baseline CD4 Counts (cells/μL)	Initial Treatment	Time to IRIS (weeks)	Clinical Presentation	Event CD4 counts (cells/μL)	IRIS Treatment	Outcome	Time to Response (weeks)
CASE I	HIV WHO stage II PPE	4	TDF/FTC/EFV Cotrimoxazole prophylaxis Cetirizine	2	Inflammatory lesions on upper limbs, face and trunk	134^a^	Fluconazole Prednisolone Cetrizine	Complete response	8
CASE II	HIV WHO Stage IV CAP Esophageal candidiasis	1	TDF/FTC/EFV Amoxicillin Fluconazole Cotrimoxazole prophylaxis	7	Inflammatory lesions on upper limbs, face and trunk	188	Griseofulvin, Prednisolone Cetrizine	Complete response	2

*CAP* community acquired pneumonia, IRIS, immune reconstitution inflammatory syndrome, *PPE* papular pruritic eruption, *TDF/FTC/EFV* tenofovirdisoproxilfumarate / emtricitabine / efavirenz

^a^CD4 measured 6 weeks after the event

### Case II presentation

A 35 years-old newly HIV-diagnosed female presented to the HIV clinic complaining of generalized body itching associated with intermittent papular rashes involving both lower limbs, which had started 1 month prior to the visit. She also had difficult in swallowing and dry cough for 1 week. She reported experienced night sweats, weight loss and chest pain for the last months. On physical examination she had oral thrush, PPE involving both lower limbs and bronchial breath sounds on auscultation. Other systems explorations were normal.

### Investigations

Her baseline CD4 count was of 1 cell/μL (0 %). Her CBC showed normocytic normochromic anemia (haemoglobin 9.8 g/dl, MCV 86 femtoliter, MCH 28 picograms/cell) with an otherwise unremarkable CBC and normal LFT and eGFR. She had negative CRAG, VDRL, HBsAg and cervical cancer screening. Her chest radiograph showed alveolo-interstitial opacities involving the left lower lobe and a diagnosis of community acquired pneumonia (CAP) was reached. Sputum smears were negative for Acid Fast Bacilli and Xpert MTB/RIF on sputum was negative as well.

### Treatment

She received fluconazole 150 mg once daily for 21 days for oropharyngeal and suspected esophageal candidiasis, amoxicillin 1 g three times daily during week one for CAP, prophylaxis with cotrimoxazole 960 mg once daily and 400 mg of ferric ammonium citrate / 3 mg of folic acid daily for 1 month for anemia. She started ART 3 days later with TDF/FTC/EFV.

### Outcome and follow-up

**Fig. 2:**
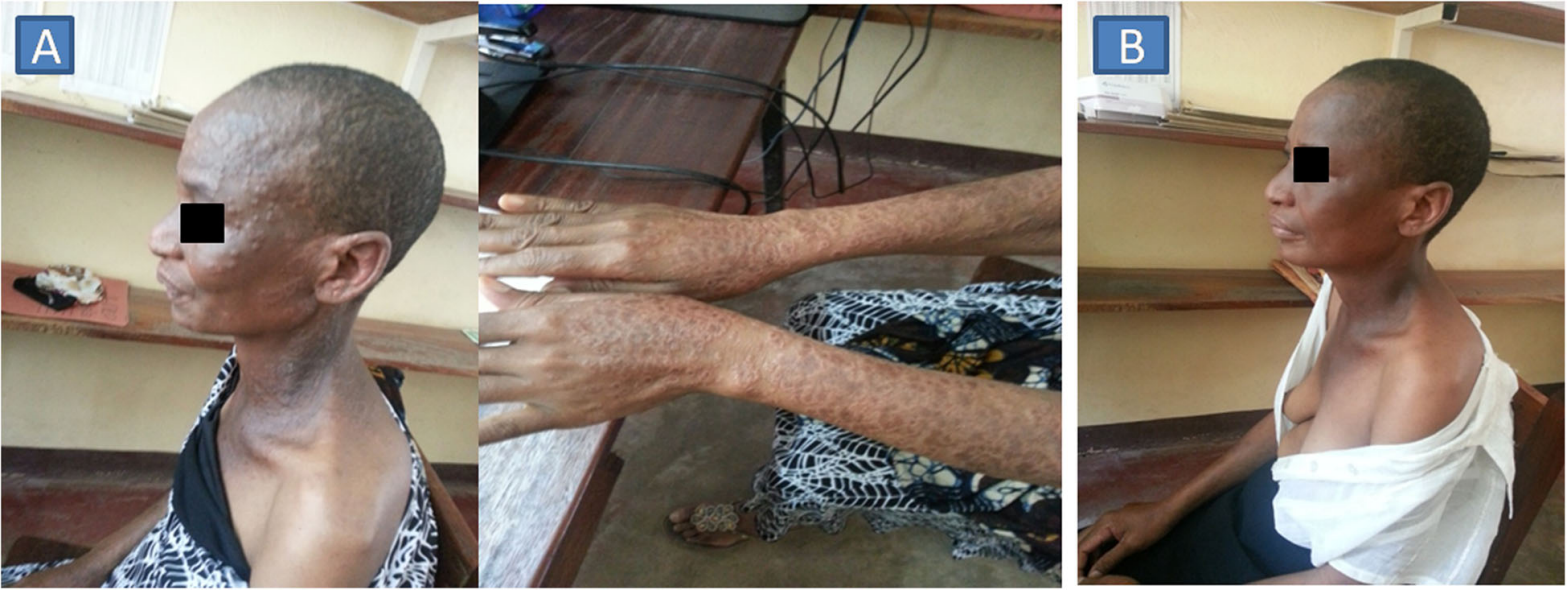
Pictures of the case II before and after treatment. Picture **a** shows inflammatory skin plaques with elevated margins and central hyperpigmentation on the trunk, face and limbs 7 weeks after ART initiation. Picture **b** shows improvement after 2 weeks treatment with griseofulvin, prednisolone and cetirizine

Seven weeks after ART initiation she started to experience skin rashes in both upper limbs, which rapidly involved the neck, trunk and the face (Fig. 2). The lesions clinically progressed to inflammatory skin plaques with elevated margins and central hyperpigmentation on the trunk, face and limbs in the frame of a good general recovery and increased CD4 count and percentage (188 cells/μL - 6 %). Tinea-IRIS was suspected and treated with griseofulvin 500 mg once daily with prednisolone 1 mg/kg for 1 week tapered down during the second week and cetirizine 5 mg once daily for 1 week. At the end of the second week of antifungal and steroid treatment, there was total resolution of the lesions (Fig. 2) (Table 1).

## Discussion

The occurrence of PPE in adult African patients has been observed as highly predictive of HIV infection [3] and has been correlated with low CD4+ cell count (<200 cells/l). Differential diagnosis of PPE includes tinea, a fungal infection which involves the keratinized epidermis, nails, and hair caused by common opportunistic pathogens associated with HIV.

No comprehensive studies on dermatophyte infections have been conducted on HIV-individuals in low-income countries (LIC), where more than 90 % of the global HIV-infected population resides. Prevalence of tinea infestation among people living with HIV (PLHIV) is likely to be high and to occur in all HIV-infected patients at one point of their lives [4]. Three types of dermatophytes account for the majority of infections: *Epidermophyton*, *Trichophyton*, and *Microsporum.* The most common dermatophytes affecting PLHIV includes *Trichophyton rubrum,* which is known to be the most prevalent, followed *by Trichophyton mentagrophytes, Trichophyton tonsurans, Candida albicans,* and *Epidermophyton floccosum* [5]*.* The infection in PLHIV has been associated with the degree of immunosuppression, being more frequent with low CD4 count like in our two patients. The clinical presentation range from being asymptomatic to disseminated disease affecting all four extremities.

Tinea, also known as ring worm, is the term representing a variety of skin mycoses and its diagnosis could be confirmed by potassium hydroxide (KOH) examination of scrapings from material taken from the active border of the lesions. This is a very simple laboratory examination which should be made widely available in resource limited settings. A fungal culture on Sabouraud’s medium can also be used to confirm the diagnosis, although it is slower. Although the etiological diagnosis relies on microbiological techniques, in rural settings of LIC diagnosis is mostly based on clinical judgment.

The availability of ART in SSA has significantly improved the quality of life for PLHIV. However, despite the fact that ART usually causes viral load suppression and restores patient’s immunity, in some patients the restored immune response is immunopathological and causes the immune reconstitution inflammatory syndrome (IRIS) [6]. IRIS occurs when immunity is restored especially in the first months of effective ART. In a prospective cohort study conducted among HIV patients in South Africa, 10.4 % of patients initiating ART developed IRIS [7]. Mucocutaneous manifestations of IRIS have been poorly documented. A study conducted in Mozambique showed 26.5 % of patients developed IRIS after a median time of 62 days from ART initiation with 53 % of them having mucocutaneous manifestations. Tinea accounted for 47 % of all mucocutaneous IRIS cases [8]. In another study conducted in Brazil, 10–25 % of unselected patients starting ART developed IRIS with 52–78 % of these patients presenting with cutaneous features [1, 9]. Tinea-IRIS lesions can be visible on patient’s skin, and despite not being associated with mortality can cause a decrease in the quality of life [10]. Moreover, it requires a high index of suspicion to be treated.

In this report, we present two HIV infected patients whom we suspected to develop tinea-IRIS after starting ART. The first case developed tinea-IRIS 2 weeks after starting ART and was successfully treated with fluconazole and prednisolone. The second case presented with suspected tinea-IRIS 7 weeks after starting ART. With the evidence of increase in resistance to fluconazole after a prolonged exposure and the fact that she was treated for esophageal candidiasis with fluconazole for 3 weeks, she was treated with oral griseofulvin and prednisolone and her symptoms subsided completely after 2 weeks of treatment. Although highly suggestive of IRIS due to the temporal association with ART initiation and the concomitant immune reconstitution, *de novo* dermatophyte infection cannot be ruled out in this case [11]. We treated both patients with two locally available antifungals which have shown to be effective against tinea infection and can be combined with steroids [2].

## Conclusion

To our knowledge these are the first two cases with tinea-IRIS to be reported in Tanzania. The two cases presented had advanced immunosuppression and developed de-novo exaggerated inflammatory lesions compatible with tinea corporis and facies in temporal association with ART initiation and good immunological response. This is compatible with unmasking tinea-IRIS, and reminds African clinicians about the importance of considering this entity in the differential diagnosis of patients with skin lesions developing after ART initiation, as fungal skin infestation is likely to be widely prevalent. Close follow-up is warranted in late presenters to timely identify and treat IRIS. In LIC like Tanzania where there are 4,993 estimated cases of serious fungal infections per 100,000 person/year, clinical judgment, appropriate microbiological tests and laboratory trained technicians are vital [12]. Additionally, since treatment with readily available in LIC antifungals such as fluconazole and griseofulvin combined with predinisolone have been proved to be effective, early diagnosis and treatment is paramount to reduce the associated morbidity, as shown by these two cases.

## Abbreviations

CAP: Community acquired pneumonia
CBC: Compete blood count
CRAG: Cryptococcal plasma antigen
eGFR: Estimated glomerular filtration rate
HBsAg: Hepatitis B surface antigen
HIV: Human immunodeficiency virus
IRIS: Immune reconstitution inflammatory syndrome
KOH: Potassium hydroxide
LFT: Liver function test
LIC: Low-income countries
MCH: Mean corpuscular hemoglobin
MCV: Mean corpuscular volume
PLHIV: People living with HIV
PPE: Pruritic papular eruptions
SSA: Sub-Saharan Africa
TDF/FTC/EFV: tenofovir/emtricitabine/efavirenz
Tinea-IRIS: Tinea-immune reconstitution inflammatory syndrome
VDRL: Venereal disease research laboratory
